# Supplementary material for: Content-rich biological network constructed by mining PubMed abstracts
Source: BMC Bioinformatics. 2004 Oct 8;5:147. doi: 10.1186/1471-2105-5-147 (PMC528731; doi:10.1186/1471-2105-5-147)
Supplement: Additional File 2 — The original results of the above study (non-essential files are deleted to keep the file size under the limit set by BMC bioinformatics). [file 1471-2105-5-147-S2.bz2 › chilibotAdditionalFile2/dip05/136ID8872303E489/html/GAP43_CALM1.html]

 


 **GAP43** and **CALM1** 
  
Found 143 abstracts in PubMed, retrieved 05.  
 

 What does Google say? 
 PDF only 
| .edu only 

---

**Interactive relationship** (e.g. stimulation, inhibition, etc)

**Inhibitory relationship**- Administration of palmitoylcarnitine, although did not change the subcellular distribution of GAP 43  [ **GAP43** ] , decreased its phosphorylation, which could regulate other signal transduction pathways calmodulin  [ **CALM1** ]  and G 0 dependent.  Ref: 12061781 Biochem Biophys Res Commun, 2002
**Neutral relationship**

**Non-interactive relationship** (e.g. studied together, co-existance, homology, etc.)

- To better understand the molecular basis of this neuroanatomical alteration, the present study examined three developmentally regulated protein kinase C substrate mRNAs that are highly expressed in hippocampal granule cells during mossy fiber outgrowth GAP 43  [ **GAP43** ] , a presynaptic substrate implicated in axonal outgrowth, RC3 neurogranin, a postsynaptic substrate implicated in calmodulin  [ **CALM1** ]  signaling, and MARCKS like protein MLP, which binds calmodulin  [ **CALM1** ]  and filamentous actin in neurons and glial cells.  Ref: 12697986 Dev Neurosci, 2002
